# Microbially Mediated Chemical Ecology of Animals: A Review of Its Role in Conspecific Communication, Parasitism and Predation

**DOI:** 10.3390/biology10040274

**Published:** 2021-03-27

**Authors:** Mónica Mazorra-Alonso, Gustavo Tomás, Juan José Soler

**Affiliations:** 1 Departamento de Ecología Funcional y Evolutiva, Estación Experimental de Zonas Áridas, Consejo Superior de Investigaciones Científicas, 04120 Almería, Spain; 2 Unidad Asociada (Consejo Superior de Investigaciones Científicas): Coevolución: Cucos, Hospedadores y Bacterias Simbiontes, Universidad de Granada, 18071 Granada, Spain

**Keywords:** bacteria, chemical communication, ectoparasite–host interaction, microbiome, predator–prey interaction, volatiles

## Abstract

**Simple Summary:**

Symbiotic bacteria and fungi facilitate the acquisition of nutrients to their animal hosts, protect them against predators, parasites and diseases, and, in some ways, modulate complex animal behavior, including communication, by means of chemical signaling. However, odors of symbiotic bacterial origin would not only inform conspecifics of their animal host, but parasites and/or predators may also use those odors to detect their victims. We here review the role of bacterial symbionts on animal communication, and on interactions of their animal hosts with parasites and predators. Moreover, because microbial symbionts can have negative effects on their hosts facilitating predation and parasitism, these enemies could modulate the microbial community of animals, and we reviewed the available evidence supporting this idea. The inclusion of microorganisms in scenarios of communication, parasitism, and predation opens up new avenues of research that will contribute to understanding such interactions. We here elaborate some predictions and provide some guidance for future research.

**Abstract:**

Microbial symbionts are nowadays considered of pivotal importance for animal life. Among the many processes where microorganisms are involved, an emerging research avenue focuses on their major role in driving the evolution of chemical communication in their hosts. Volatiles of bacterial origin may underlie chemical communication and the transfer of social information through signals, as well as inadvertent social information. We reviewed the role of microorganisms in animal communication between conspecifics, and, because the microbiome may cause beneficial as well as deleterious effects on their animal hosts, we also reviewed its role in determining the outcome of the interactions with parasites and predators. Finally, we paid special attention to the hypothetical role of predation and parasitism in driving the evolution of the animal microbiome. We highlighted the novelty of the theoretical framework derived from considering the microbiota of animals in scenarios of communication, parasitism, and predation. We aimed to encourage research in these areas, suggesting key predictions that need to be tested to better understand what is one of the main roles of bacteria in animal biology.

## 1. Introduction

Interactions between animals and their associated microorganisms (i.e., microbiota) are nowadays considered of pivotal importance to understand the physiology, morphology, and behavior of animals, as well as the outcomes of their interactions with abiotic and biotic environmental conditions [1]. Beyond pathogenesis, the most commonly studied effects of microorganisms on animals are those that link the gastrointestinal microbiota with facilitation of nutrient absorption, or even the synthesis of some essential micronutrients [2,3,4]. During the last two decades, the scientific interest has begun to consider the microbiota as an essential component of living animals, therefore affecting their evolution [5]. An emerging topic in evolutionary biology deals with the importance of the microbiome in mediating communication in their host organisms [6].

Animals acquire information from the environment by direct interactions in trial-and-error-tactics (personal information), or by monitoring the interactions of others with the environment and their outcomes, thereby acquiring what is called social information (SI) [7]. Social information can be based on signals, which are traits that specifically evolved to convey information to receivers [8]. Alternatively, social information can also be based on cues provided inadvertently by individuals while engaged in their biological activities (inadvertent social information, ISI) [7]. Signals usually inform or advertise receivers on the phenotypic condition and capabilities of the sender, which supposedly benefits both sender and receiver [8,9]. ISI may inform bystanders, for instance, about resource location, but also about the quality of the resource, which is revealed by the performance or phenotypic quality of the cue sender (i.e., public information) [7]. Importantly, signals, as well as ISI, are supposed to reliably convey information on the phenotypic condition of the sender. Honesty of signaling characters has mainly relied on the hypothesis that only high-quality individuals will be able to afford its associated costs [8,10,11]. Instead, ISI is supposed to convey information on the phenotypic quality of the sender as a result of individual performance [7].

Depending on the type of the sensitive channel used to transmit the signal or to gather ISI, stimuli have been mainly classified as visual, auditory, or chemical. The use of chemicals is the most ancient, widespread, and shared way used by living organisms to evaluate their environment and to communicate with each other [12]. Remarkably, symbiotic bacteria, or what as a whole is known as microbiota, are largely responsible for animal scents [13,14]. The role of symbiotic bacteria in animal chemical communication is therefore paramount [6]. Moreover, the microbiota is intimately related to the phenotypic quality and physiological activity of their animal hosts [15,16,17] by influencing their growth and development [1]. This indeed will affect characteristics of signals and ISI that conspecifics and heterospecifics could use. Particularly interesting is the possibility that chemical signals and cues of bacterial origin can be eavesdropped on by unintended receivers, such as parasites and predators, when locating and selecting hosts and prey.

Yet symbiotic microorganisms may also influence the outcomes of the interactions between their hosts and their hosts’ enemies (predators and parasites) in other ways. For instance, microorganisms largely determine host health and condition [1,3], and these effects could be also used by predators and parasites as inadvertent social information that facilitate host detection and/or selection [14]. Symbiotic microorganisms can also produce metabolites with antimicrobial properties [18] that clear or prevent parasitic infections. Some bacterial symbionts are also known to produce metabolites that deter predators or parasites [19]. Symbiotic microorganisms might even be related to adaptive hormonal and immunological plastic responses of hosts against stressful environmental conditions, including those related to the risk of parasitism or predation [20]. All these possibilities highlight the hypothetical role of microorganisms in driving the interaction between hosts and their parasites and predators, and we have here reviewed current knowledge on these matters.

**Figure 1:**
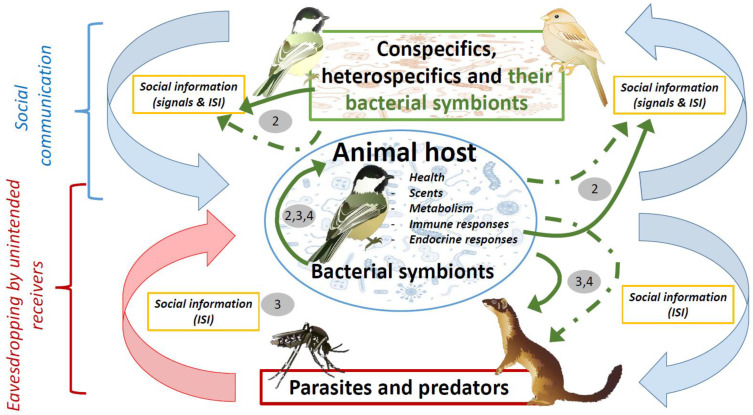
Diagram showing hypothetical influence of bacterial symbionts (green arrows) in scenarios of social communication, parasitism, and predation. These influences could be directly due to either bacterial metabolism or products with antimicrobial or antipredatory properties (solid arrows), or indirectly through their effects on host characteristics (i.e., health, scents, metabolism, immunity, and hormones) (dashed green arrows). Bacterial symbionts contribute to social information that is received by conspecifics or heterospecifics, including parasites and predators. The negative effects of parasites and predators (red arrow) would be directly counteracted by defensive products of bacterial origin, or indirectly by host defensive traits that are also influenced by bacteria (continuous and dashed green arrows connecting the host with parasites and predators). These negative effects however will be enhanced by eavesdropping on inadvertent social information directly or indirectly mediated by host microbial symbionts and, thus, parasites and predators will also influence the symbiotic association between animals and microorganisms. Pathogenic parasites could also influence health and, consequently, bacterial symbionts of their victims, and, thus, parasites could indirectly affect conspecific communication. Numbers refer to main sections in the text where that relationships are covered. Symbols courtesy of the Integration and Application Network, University of Maryland (ian.umces.edu/symbols/) and freepik.com (accessed on 5 February 2021).

The role of microbial symbionts on animal chemical communication has been reviewed several times during the last decade [6,14,21] and was not the aim of this essay. Here, we rather sought to explain the rationale behind the social information value of volatiles of microbial origin in scenarios of animal communication. We also aimed to formulate key predictions to assess this hypothetical role of bacterial symbionts, and discuss the importance of bacterial symbionts in the evolution of conspecific communication, and in host–parasite and prey–predator interactions. The animal microbiome may cause beneficial as well as detrimental effects to its host. Special attention has been paid to the possibility that host enemies might eavesdrop on inadvertent social information mediated by beneficial microbiotas from their victims. An overview of the potential interactions between hosts and their bacterial symbionts in scenarios of social communication, parasitism and predation that are dealt with in this essay is shown in Figure 1.

## 2. Conspecific Chemical Communication Mediated by Bacterial Symbionts

The hypothetical role of microorganisms in animal communication is rooted in the “fermentation hypothesis”. This hypothesis was originally formulated to explain the odors of anal sac secretions of cats and foxes in the 1970s [22,23], but is now applied to the general odor profile of animals that could operate in a large variety of scenarios of olfactory communication [6,21,24,25]. Until very recently, microbial production of chemical signals had been mainly described in mammals and insects [6]. However, solid evidence for the role of bacterial symbionts in producing volatile metabolites that contribute to the host odor profile is rapidly being accumulated for a wider range of animal taxa, including not only mammals [15,16] and insects [14,26,27], but also amphibians [28] and especially birds [17,29,30].

Evidence supporting the key role of bacterial symbionts in animal communication represents one of the most fascinating and important advances that chemical ecology has experienced during the past few years [21]. The role of bacterial symbionts in animal communication is based on the assumption that some genetically and environmentally determined characteristics of animals, like those related to diet and immunity, also determine their microbial symbionts [31,32]. Consequently, volatiles of microbial origin would inform the characteristics of their animal hosts (social information), and therefore, these chemicals would contribute to olfactory communication [6]. For instance, because of the many factors determining animal microbiotas, and the enormous variability of associated microbial volatiles, the particularities of chemical profiles due to the metabolism of bacterial symbionts may communicate individual identity to conspecifics (i.e., an individual signature) [21,33]. Microbial volatiles may also aid in easy navigation towards nest locations for parents or offspring due, for instance, to the particular volatile profile of feces around nests [34,35]. Also, in scenarios of parent–offspring communication, volatiles from symbiotic bacteria might help newborn mammals to recognize their own mother’s nipples [36,37], while in sexual selection scenarios these volatiles can be used to choose a genetically compatible partner [38,39].

Previous research on this topic has been mainly focused on the possibility that volatiles derived from the metabolisms of host bacterial symbionts function to assess host quality by conspecifics. As we mentioned before, the microbiota composition, and thus volatile profiles of bacterial origin, depends on host characteristics, which include components of the host phenotypic quality. Thus, because these volatiles would convey valuable information to conspecifics, selection will favor receivers using such cues of microbial origin [31]. However, to demonstrate that microorganisms convey information on the phenotypic characteristics of their hosts, linking particular volatiles predicting host characteristics with the microorganisms that produce such volatiles is needed. Exploring associations between microbiotas and the odor profiles of the hosts reflecting their physiological characteristics is nowadays a fruitful area of research that will help to unveil general patterns on the role of microbiotas in social communication. Metagenomics, together with other omic techniques studying the metabolic production of microbial communities (e.g., proteomics and metabolomics), will help to characterize the microorganisms responsible for the production of particular volatile metabolites [40], allowing us to fill important gaps in our knowledge regarding the role of the animal microbiome in chemical ecology and communication.

The evolution of chemical signals mediated by microbial symbionts requires, not only that those odors reliably reflect characteristics of their hosts, but also that receivers use the conveyed information, and that such communication benefits both sender and receiver. By providing microorganisms with substrates in special locations such as the gut or glands, animals of several taxa cultivate bacteria, producing substances that are valuable for them in terms of micronutrient provisioning, antimicrobial defenses, or even signaling [1,41,42]. Scents originating from symbiotic microorganisms that inhabit animal glands are particularly important to gain insight into the evolution of microbially mediated chemical communication. This is mainly because most scents derived from animal glands have been traditionally considered as classical examples of chemical signals. Evidence supporting the existence of microbial symbionts growing within such scent glands are being accumulated in the literature, especially in exocrine glands, such as the anal glands of mammals [15,16] and the uropygial glands of birds [17,30]. Similarly, scents derived from microorganisms that enhance the survival and reproductive success of their hosts, such as those from gut microbiotas, would also signal host phenotypic quality, therefore evolving into an associated signaling role. Consequently, in these cases, where scents of microbial origin are part of the animal chemical signaling, the evolution of chemical communication should entail changes not only in genetically inherited characteristics of hosts, but also in characteristics of their microbial symbiotic communities. However, changes in animal hosts and in their microbiotas should not be seen as completely independent, because animal characteristics will largely determine characteristics of their microbiotas.

Even though it is generally assumed that the fitness of the host is often linked to that of its microbiota [43,44], but see [45], the evolution of symbiotic bacterial communities by means of natural selection acting on hosts entails important theoretical challenges. This is mainly because the characteristics of microbial symbionts are not directly determined by animal genomes and, thus, natural selection acting on host performance or fitness would not be able to directly modulate the bacterial community of symbionts, nor their chemical profiles. To overcome this theoretical problem for explaining the evolution of host microbiomes, some authors have claimed that microbiomes and individual hosts should be considered together as the unit (holobiont) where natural selection acts [46,47,48]. It has been broadly recognized that the characteristics and composition of host-associated microbial communities parallel the phylogeny of the related host species, and the holobiont concept would a priori help to understand the evolution of such phylo-symbiosis [49]. This approach, however, may entail some other theoretical problems related to group selection theory [50]. A main critique to the holobiont concept is that fitness of hosts and symbionts are not fully linked, especially not for all members of a host-associated microbiota [45]. Thus, alternative scenarios explaining phylo-symbiosis have been proposed and explored. One possibility is to consider genetically determined traits in animal hosts that allow or favor maintenance of certain microbial communities, driven, for instance, by host variability in diet or habitat. The allelic variation in genes determining such traits will therefore predict the composition of, or functional variation in, microbiotas [51]. In this case, host characteristics determining, for instance, the mode of transmission of bacterial symbionts and the characteristics of the environment where microbes are hosted will also govern the composition of symbiotic bacterial communities and their metabolic activity [52]. Therefore, it is possible that natural selection acting on hosts could determine the metabolic activity of the microbiota, including those microorganisms responsible for the production of volatiles with importance in chemical communication. Similarly, for visual traits, we know, for instance, that the eggshell coloration of hoopoes (*Upupa epops*) is affected by uropygial secretion rubbed on eggshells by females during incubation, which is indeed mediated by symbiotic bacteria hosted in the uropygial gland [53]. The eggshell color functions as a signal of female quality [54], while the symbiotic bacterial community hosted in the uropygial gland of hoopoes have a significant genetic component [55,56]. Thus, because characteristics of the microbial community of the uropygial secretion are likely mediated by physiological characteristics of hosts (where natural selection can operate), natural selection processes would also be responsible for egg coloration in hoopoes.

Similar processes to those described above for hoopoes can also operate for olfactory traits mediated by symbiotic microorganisms. Host characteristics that enhance the establishment of the microbiota with direct beneficial effects for hosts could be identified by characteristic host odors. Moreover, because of the potentially narrow link between those host traits and characteristics of the volatile profile of the associate microbiota, host odor mediated by bacterial symbionts will also reflect host characteristics favoring the establishment of particular microbiotas. Thus, the effects of natural selection acting of host traits could easily be tracked by following variation in host odor profiles. Interestingly, because mating with individuals with characteristics that enhance growth of beneficial microorganisms would be of selective advantage, sexual selection acting on olfactory traits mediated by bacterial symbionts will also accelerate the evolution of these characteristics. Future research should focus on identifying (i) physiological or morphological host traits enhancing the establishment and growth of beneficial microbiotas, (ii) characteristic microbial volatiles narrowly reflecting potential fitness effects for their hosts, and (iii) whether sexual selection favors hosts of particular bacterially mediated odor profiles. These research will allow to gain insight into the mechanisms underlying the evolution of hosts characteristics that favor particular microbiotas.

Microbial symbionts would also contribute to the inadvertent social information provided by their hosts. Interestingly, this information does not necessarily benefit hosts, but reliably informs conspecifics and heterospecifics about host phenotypic characteristics or condition. It may be the case that pathogens, or parasite infections, influence the host microbiota, which would result in animals displaying particular volatile profiles. Conspecifics could thus use that ISI as a warning chemo-sensory signal to, for instance, avoid close contacts with sick individuals. For example, in humans, experimental activation of the immune system affected body odor, which was judged by conspecifics as less pleasant, more intense, and less healthy [57]. Similarly, mice, mandrills, and lobsters are able to identify sick conspecifics via chemical cues [58,59,60,61]. Thus, the role of bacterial symbionts in indirectly mediating communication of health status to conspecifics could be widespread among animals. Importantly, emitting such volatiles might have negative effects on their hosts. Therefore, there may be evolutionary processes to selecting host traits that shape the microbiota in emitting volatiles with less detrimental effects. Future work should also explore the role of bacterial symbionts as producers of inadvertent social information with detrimental effects for their hosts.

## 3. Negative Effects of the Microbiome in Relation to Parasitism and Predation

Social information derived from the metabolism of host microbial symbionts can be eavesdropped on by unintended receivers such as parasites and predators. These two actors can substantially mediate some of the negative effects promoted by conspecific social communication, even when this information benefits both senders and conspecific receivers. Predation and parasitism are among the most powerful natural selection forces driving the evolution of animals in general and of animal signaling in particular [62,63,64,65]. Examples of predators and parasites eavesdropping on auditory or visual cues from their victims are relatively well known [11,64,66,67,68,69]. Paramount examples include the adaptive disappearance of song in crickets due to parasitoids eavesdropping on this sexual signal [70], or how frog-eating bats influence the evolution of frog calls [71]. The possibility that symbiotic microorganisms can also mediate the interactions that parasites and predators maintain with their victims opens a more complex picture for the evolution of chemical communication systems (Figure 1). Below we describe some particular scenarios that could exemplify this possibility.

Many microorganisms cause animal disease. Apart from the fact that sick animals typically downregulate their antiparasitic and antipredatory defenses [72,73,74], their microbiomes typically differ from those of healthy animals [75]. Thus, the effects of disease on the probability of predation and parasitism could also be mediated by changes in the microbial volatile profiles of sick animals that are detected by parasites and predators. This is apparently the case of those volatiles emitted by the bacteria that colonize the wounds of hot-blooded vertebrates, which attract parasitic flies that lay their eggs or larvae on infected or necrotized wounds [76].

Further examples of symbiotic bacteria indirectly mediating host detection by enemies come from research on host preference by mosquitoes and related ectoparasites [77]. Although parasites may directly induce behavioral changes in their hosts that are aimed at increasing parasite transmission [78], preferences by mosquitoes toward odors of already parasitized hosts are likely mediated by changes in host microbiotas [79,80]. Hematophagous insects acting as vectors of human diseases (e.g., malaria, yellow fever, dengue) [81,82,83,84,85] might exemplify this possibility. It has been suggested that mosquitoes may be more attracted to the odor of *Plasmodium*-infected humans and birds [72,73], but see [77], and some evidence suggests that bacteria play key roles in determining host preference by mosquitoes. For example, laboratory experiments have revealed that the mosquito *Anopheles gambiae*, a main malaria vector in humans, is more attracted to individuals whose skin bacterial community is less diverse, but more abundant, and that includes *Staphylococcus epidermis* [82]. Similarly, humans whose skin contains more diverse volatile profiles were less susceptible to *Aedes aegypti* bites [85]. Furthermore, other components of the human skin bacterial community, such as *Pseudomonas* spp. and *Variovorax* spp., seem to be the key taxa explaining why some individuals are unattractive to mosquitoes [82]. Interestingly, in trying to link the characteristics of the bacterial community with those of the chemical profiles of human skin that influence host selection by mosquitoes, Verhulst and coauthors [81] tested the effects of volatiles from six species of bacteria obtained from the cultivars of human skin on host preference by *A. gambiae*. They found this mosquito was not attracted to *Pseudomonas aeruginosa* and its blend of volatiles, while it was attracted to a blend of volatiles from *Corynebacterium minutissimum*, *S. epidermis*, and *Bacillus subtilis*. Taken together, these results suggest that certain volatiles of bacterial origin can facilitate parasitism, while others can deter enemies (see next section). In addition, outside of blood-sucking ectoparasites, it has been demonstrated that during oviposition and feeding behavior, *Drosophila melanogaster* and dung beetles show an aversion towards volatiles such as phenol, which is produced by harmful bacteria [86]. Most of this research has been carried out under laboratory conditions, or was focused on particular groups of bacteria species. Future work should therefore expand on detecting particularities of the complete animal microbiota associated with the production of chemicals affecting host selection by parasites. Furthermore, detecting such associations is necessary to ascertain the role of the whole microbial symbiotic community in determining host chemical profiles and host preference by blood sucking ectoparasites.

Field experiments have also revealed that the symbiotic bacteria inhabiting avian nests impinge on the risk of predation and parasitism. For instance, manipulation of the bacterial community of avian nests affected development and survival prospects of great tit (*Parus major*) [87] and spotless starling (*Sturnus unicolor*) nestlings [88]. Interestingly, the microbiota of great tit nest materials and feathers determined the chemical volatiles released from their nests [89], which therefore could affect the probability of parasitism and predation. In accordance with this possibility, the experimental breakage and delivery of fecal sac contents of spotless starling nestlings increased the bacterial density in their nests, and was related to an increased predation rate and ectoparasite load [90]. Similarly, nestling hoopoes developing in nest boxes where microorganisms were experimentally eliminated from nest substrates suffered reduced ectoparasite loads [91]. These results, therefore, suggest that selection pressures due to parasitism and predation should influence the evolution of host characteristics allowing the establishment of particular microbiotas that produce volatiles, which would reduce the risk of being detected by enemies. This interesting possibility could be tested by performing experiments known to influence both the bacterial community of avian nests and the risk of parasitism experienced by nestlings (e.g., breakage of nestling feces, addition of feathers or aromatic plants to the nest, or autoclaving nest material before reproduction [90,91,92,93,94]). Differences in risk of parasitism experienced by nests under different experimental treatments should covary with differences in microbiotas and volatile profiles. Moreover, this hypothesis can also be tested by exploring whether prevalence and abundance of particular bacteria and volatiles known to affect detection of potential victims by parasites and/or predators differ in populations under different risk of predation or parasitism. Finally, experiments in laboratory conditions during several generations of hosts, directed to explore expected evolutionary changes in volatile profiles and microbiota compositions in relation to parasitism or predation risk, could be also performed to test the role of animal enemies determining microbiotas and associated chemicals.

All these previous examples support potential negative effects of microbial symbionts of hosts, due to parasites or predators eavesdropping on host volatiles of bacterial origin. However, because parasites and predators also emit volatiles of microbial origin, it is equally plausible that these volatiles have negative effects on predators and parasites because their victims could use this inadvertent social information to reduce parasitism or predation risk. When victims receive ISI of the presence of parasites or predators, they would be able to display antiparasitic or antipredator defenses. Especially well known is the case of predators whose chemical cues can reveal their presence to potential prey of different taxa [95,96,97]. Thus, volatiles produced by microbial symbionts could have negative effects for their hosts independently of the side of the antagonistic interaction where these hosts are (i.e., prey or predator, host or parasite). These negative effects of microbial symbionts in terms of host detectability would occur independently of whether the symbiotic association qualifies as mutualistic when interactions with undesired receivers of ISI are not considered. Thus, to understand the net cost/benefit balance of the symbiotic associations between microorganisms and their animal hosts, negative effects derived from natural enemies eavesdropping on bacterial derived volatiles should be considered. We are only starting to comprehend the role of volatile-producing microbial symbionts on scenarios of parasitism and predation and more research dealing with possible costs and benefits for either parasites, predators, or victims is urged in this matter. Particularly interesting is the possibility that the strength of those ecological interactions affects the fitness outcome of associations between animals and their microbial symbionts.

## 4. Beneficial Effects of the Microbiome in Relation to Parasitism and Predation

In this section, we first describe some benefits of microbial symbionts in scenarios of parasitism and predation and, thereafter, we focus on those possibly mediated by volatiles. Beneficial effects of microorganisms on animal health are well established. This is particularly the case for gut microbiotas, mainly because nutrient absorption [3] and essential, otherwise inaccessible, micronutrients [2], including their direct syntheses [4], entirely depend on bacterial symbionts. It is also well known that unhealthy animals, or those in suboptimal physical condition, are more heavily parasitized and predated [98,99]. Thus, because animal health largely depends on their gut microbiotas, an indirect general effect of the gut microbiota of healthy animals would be a reduced risk of predation or ectoparasitism [100]. As far as we know, the relation between gut microbiota composition and probability of ectoparasitism or predation has never been directly tested.

Moreover, gut microbiotas of animals could also partially drive adaptive plastic responses to parasites and predators of their hosts [101,102]. These include hormonal [103,104,105,106] and immunological responses [107,108], allowing hosts to face parasitic and pathogen invasions. It is broadly accepted that gut microbiotas directly communicate with the animal brain through the production of some metabolites that activate the vagus nerves, or by inhibiting other nerves within the gastrointestinal system that indirectly influence the signaling of various mediators to the brain [20]. In both cases, gut microbiotas would be involved in the mechanisms determining the social behavior of hosts [20] and, thus, their exposure to parasites and predators. This theoretical background therefore suggests that, through different pathways, gut microbiotas might drive endocrine, immune, and behavioral responses to the risk of parasitism and predation. To the best of our knowledge, this possibility has never been tested and, therefore, opens a new interesting research avenue about the role of microbial communities on the antiparasitic and antipredatory responses of animals.

For defense against predator or parasite enemies, some animals use metabolites synthesized by other organisms with antimicrobial and antipredatory properties (i.e., self-medication [94,109,110,111,112,113]). Animals also use defensive metabolites that are endogenously produced (e.g., uropygial gland secretions of birds [113,114,115,116,117]). Some of these endogenously produced compounds are volatiles, and abundant correlative and experimental evidence supports the role of such metabolites in interfering with host attractiveness to predators and parasites [85,118,119,120,121,122]. Interestingly, evidence of bacteria living within exocrine glands of animals is accumulating [15,16,17,22,23,29,30] and, thus, some of these endogenously produced chemicals may have a bacterial origin, a possibility that has been scarcely explored. The role of bacteria in the production of these chemicals is a recently opened line of research with promising future possibilities, and different studies have provided evidence for these mutualistic associations in different taxa [41,123,124,125,126].

Examples of bacteria producing metabolites that defend their hosts against predators or parasites are abundant in invertebrate animals [19]. For instance, *Wolbachia* and *Spiroplasma*, two phylogenetically widespread parasitic endosymbionts of insects, enhance resistance of hosts against a variety of viral diseases [127], parasitic nematodes [128], and parasitic wasps [129]. Antibiotic producing symbionts are also known for several groups of insects including for instance digger wasps [130], fungus-growing ants [131], and pine beetles [132]. Other insects benefit from the production of toxins or antibiotics by microbial symbionts that reduce palatability or prevent pathogenic infections. For instance, the gut microbiota of coccinellid beetles produces antipredatory volatiles (i.e., methoxypyrazines) [27]. Similarly, endosymbionts of some species of rove beetles of the genus *Paederus* produce toxins that deter wolf spiders [133]. Interestingly, some of these chemicals with protective functions against certain parasites or predators could as well be detected by conspecifics, and inform them on defensive capability of emitters, or by other different parasites or predators, influencing host detectability. 

Research on these types of three-way interactions [134] that include not only the bacterial symbionts and their animal hosts, but also conspecifics and heterospecifics, can be approached in the wild by the system composed by birds, their symbiotic bacteria, and their ectoparasites (or predators). Birds possess a unique exocrine gland responsible for most avian odors and for the production of topically applied defensive metabolites, the uropygial gland [114], which allows researchers to focus studies on this special organ. In fact, groundbreaking evidence for this three-way interaction came from birds using their uropygial secretion, which contains microbial symbionts with antimicrobials and/or antipredatory properties that also might repel ectoparasites. This is, for instance, the case of green woodhoopoes (*Phoeniculus purpureus*) hosting symbiotic bacteria in their uropygial glands, which produce metabolites that repel predators [29,135]. Similarly, the uropygial secretions of nestling hoopoes also include bacterial symbionts that produce antimicrobials [136,137,138] and repellents for mosquitoes and biting midges [113]. Associations between characteristics of the uropygial gland and the risk of parasitism [139,140,141], as well as symbiotic bacteria living in the uropygial gland [17,142,143,144], are now described in phylogenetically distant bird species. Thus, the beneficial effects of uropygial secretions for birds may be mediated by microbial symbionts. This is a field worth being explored, and that may allow researchers to detect new undescribed mutualistic relationships between bacteria and their avian hosts. In the next section, within the final remarks, we review current knowledge on the possibility that parasites and predators modulate beneficial effects of volatile-producing bacteria for their animal hosts.

## 5. Final Remarks

We have reviewed the state of the art on the role of microbial symbionts on animal communication, paying special attention to chemical communication with conspecifics, and to the interactions between their hosts with parasites and predators. These two issues converge on the possibility that parasites and predators use volatiles of bacterial origin to detect and select their victims, highlighting the possibility that it occurs because, independently of their function, volatiles of symbiotic bacterial origin are in fact host signals or inadvertent social information that can be eavesdropped on by conspecifics and heterospecifics (see Figure 1).

We have discussed the importance of such interactions in the evolution of conspecific communication and host defensive traits. A wide variety of chemicals are involved in animal communication [145] and, similarly to other signals, these may be costly in terms of energy consumed or may increase the risk of parasitism and/or predation [8,146,147]. Moreover, inadvertent social information mediated by chemical volatiles, associated for instance to animal respiration (e.g., [148]) or other biological activities, reliably inform predators and parasites on the location and on the phenotypic condition of their victims. Most blood-sucking ectoparasites and predators have evolved a highly developed olfactory system that, among other functions, allows them to locate potential hosts and/or prey [121,149,150,151]. Thus, similarly to the effects described for auditory cues (e.g., [152,153], both predators and parasites should have shaped the evolution of traits related to volatile production in their victims. That would be the case independently of whether volatiles are partially produced by bacterial symbionts.

Interestingly, some volatiles of bacterial origin that parasites and/or predators eavesdrop on to detect their victims may have beneficial effects for the hosts in scenarios other than parasitism and predation. In these cases, costs imposed by eavesdropping parasites or predators would counteract possible benefits and, thus, would modulate the production of such chemicals, or even the mutualistic symbiotic association between hosts and the bacteria producing such chemicals. Exploring the chemical profiles of animal microbiotas, particularly those of bacteria producing antimicrobials or compounds linked to protection against predators, is necessary to understand these complex interactions. One possibility for hosts to reduce negative effects of parasites and predators on mutualistic associations is to recruit new bacterial strains that are somehow able to mask host cues [21], or even to produce volatile compounds that repel parasites and/or predators. Mechanisms to regulate or control bacterial growth rate, and thus the emission of volatile compounds, would also provide selective advantages [81]. Future research focused on exploring these possibilities will clarify the effects of parasites and predators on mutualistic associations between animals and bacteria on the one hand, and how hosts minimize eavesdropping by their enemies on volatiles produced by mutualistic symbionts.

Finally, it is important to highlight that a better understanding of those interactions between hosts and parasites that are mediated by chemicals produced by bacterial symbionts may have ample applications in veterinary and medical research. Most ectoparasites are vectors of important human and animal diseases. Discovering connections between the animal microbiome and the volatiles that attract or deter ectoparasites may aid in the development of new products or methods for protection against ectoparasites and the diseases they transmit [76,77]. Besides health concerns [154], parasites cause great economic losses [155,156]. Microbially derived products might substitute, or at least complement, the use of insecticides, which produce undesired side effects [157,158] or induce resistance in ectoparasites due to inappropriate and prolonged use of these drugs [159,160]. The possibility of finding natural products that might be applied without side effects is therefore an asset that should encourage further research on the role of bacteria mediating the interaction between parasites and their animal hosts.

## Data Availability

Not applicable.
